# Comparative profiling of canonical and non-canonical small RNAs in the rice blast fungus, *Magnaporthe oryzae*

**DOI:** 10.3389/fmicb.2022.995334

**Published:** 2022-09-26

**Authors:** Hyunjun Lee, Gobong Choi, You-Jin Lim, Yong-Hwan Lee

**Affiliations:** ^1^Department of Agricultural Biotechnology, Seoul National University, Seoul, South Korea; ^2^Interdisciplinary Program in Agricultural Genomics, Seoul National University, Seoul, South Korea; ^3^Research Institute of Agriculture and Life Sciences, Seoul National University, Seoul, South Korea; ^4^Center for Fungal Genetic Resources, Seoul National University, Seoul, South Korea; ^5^Plant Genomics and Breeding Institute, Seoul National University, Seoul, South Korea; ^6^Plant Immunity Research Center, Seoul National University, Seoul, South Korea; ^7^Center for Plant Microbiome Research, Seoul National University, Seoul, South Korea

**Keywords:** sRNA, Dicer, ERI-1, *Magnaporthe oryzae*, canonical RNAi, non-canonical RNAi

## Abstract

RNA interference (RNAi) is divided into canonical, Dicer-dependent and non-canonical, Dicer-independent pathways according to Dicer protein dependency. However, sRNAs processed in a Dicer-independent manner have not been reported in plant pathogenic fungi, including *Magnaporthe oryzae.* We comparatively profiled the Dicer-dependent and -independent sRNAs of *M. oryzae*. Dicer-dependent sRNAs were 19–24-nt in length, had low strand-specificity, and showed a preference for uracil at the 5′-end. By contrast, Dicer-independent sRNAs presented irregular patterns in length distribution, high strand-specificity, and a preference for cytosine at the penultimate position. Dicer-dependent sRNA loci were mainly associated with LTR-transposons, while Dicer-independent sRNAs were associated with protein-coding genes and transposons. We identified *MoERI*-1, a non-canonical RNAi component, and profiled the sRNA and mRNA transcriptomes of Δ*Moeri-1* at the mycelia and conidiation stages, as the mutant showed increased conidiation. We found that genes involved in conidiation and cell cycle were upregulated by *MoERI-1* deletion. Furthermore, a comparison between sRNA and mRNA transcriptome revealed that MoERI-1-dependent sRNAs mediate the regulation of gene expression. Overall, these results showed that *M. oryzae* has non-canonical RNAi pathways distinct to the Dicer-dependent manner and exploits MoERI-1-dependent sRNAs to regulate the conidiation process.

## Introduction

Small non-coding RNAs (sRNAs) are the core component of RNA interference (RNAi), mediating gene regulation at the post-transcriptional and transcriptional levels (Buhler and Moazed, 2007; Ghildiyal and Zamore, 2009). In the canonical RNAi pathway, a double-stranded RNA (dsRNA)-specific ribonuclease Dicer cleavages hairpin RNA or dsRNA into sRNAs. These sRNAs are loaded into Argonaute (AGO) protein to form RNA-induced gene silencing complex (RISC), which mediates the degradation of target RNAs or translational repression (Drinnenberg et al., 2009). sRNA species generated by canonical RNAi are generally categorized as siRNA and miRNA according to their sources and related RNAi components.

Since quelling, the first example of RNAi in fungi was reported in *Neurospora crassa*, canonical RNAi has been extensively studied in fungi as well as in animals and plants. Quelling and meiotic silencing of unpaired DNA (MSUD) are sRNA-based genome defense mechanisms reported in *N. crassa*. In *Schizosaccharomyces pombe*, heterochromatic siRNA (het-siRNA) processed by Dcr1 guides the RNA-induced transcriptional silencing (RITS) complex to the nascent target RNA. The guided RITS complex recruits chromatin modifying enzymes to methylate lysine 9 of histone 3, leading to heterochromatin formation (Billmyre et al., 2013). Exonic-siRNAs (ex-siRNA), which require Dicer enzymes for biogenesis, regulate the developmental processes in *Fusarium graminearum*, *Mucor circinelloides*, and *Trichoderma atroviride* (Nicolas et al., 2010; Carreras-Villasenor et al., 2013; Son et al., 2017). Fungi with a number of transposons typically have defense systems to limit transposon and retrotransposon activity via quelling-like RNAi pathways, mediated by transposon-derived siRNAs (Torres-Martinez and Ruiz-Vazquez, 2017).

Non-canonical pathways in which Dicer proteins do not participate have been reported to be responsible for the biogenesis of specific sRNAs. Piwi-interacting RNAs (piRNAs), which silence transposons in germ line cells, require AGO proteins and their catalytic activity, instead of Dicers (Siomi et al., 2011). In *Arabidopsis*, siRNAs independent of DCLs (sidRNAs) interact with AGO4 and 3′–5′ exonucleases trimming sidRNAs to the proper size, and were proposed to initiate the *de novo* DNA methylation of transposons (Ye et al., 2016). *S. pombe* also exploits a 3′–5′ exonuclease, Triman, to generate primal small RNAs (priRNAs) that trigger the positive-feedback loop of siRNA generation and heterochromatin assembly (Marasovic et al., 2013). In *N. crassa*, Dicer-independent siRNAs (disiRNAs), derived from both strands of the genome, contribute to transcriptional silencing of target genes including *FRQ* (Lee et al., 2010). *NcERI-1* is responsible for the generation of disiRNAs derived from disiRNA loci where convergent transcriptions occur. Unlike ERI-1 of *Drosophila*, human, mouse, and *S. pombe*, which binds to dsRNAs via the SAP domain, *NcERI-1* recognizes the 3′ end G-rich motif of single strand RNAs via an RNA recognition motif (RRM) and zinc-finger domain (Thomas et al., 2014; Dang et al., 2016).

Advances in next-generation sequencing and bioinformatics have enabled massive profiling of sRNAs in fungi. sRNAs with roles in genome defense against viruses and transposable elements have been profiled in fungi (Nicolás and Ruiz-Vázquez, 2013; Torres-Martinez and Ruiz-Vazquez, 2017). Possible roles of sRNAs have also been suggested in responses to exogenous stimuli under various environmental conditions in *Aspergillus flavus* (responses to water activity and temperature) and *M. circinelloides* (resistance to antifungal drug) (Bai et al., 2015; Calo et al., 2017). Profiling of sRNAs during host-pathogen interactions has revealed that sRNAs function not only as endogenous regulators but also as effectors in *Botrytis cinerea*, *Verticillium dahliae*, and *Puccinia striiformis* (Weiberg et al., 2013; Wang et al., 2017).

*M. oryzae* is the causal agent of rice blast, which causes severe yield losses in cultivated rice worldwide. Due to the scientific achievements in both fungus and the host, and global economic importance, the interaction between *M. orzyae* and rice has been a model pathosystem for studies on molecular basis of pathogenesis (Dean et al., 2012). *M. oryzae* has two Dicer-like proteins (MoDCL1 and MoDCL2), three AGO proteins (MoAGO1, MoAGO2, and MoAGO3), and three RNA-dependent RNA polymerases (MoRdRP1, MoRdRP2, and MoRdRP3). MoDCL2 plays main role in hairpin-RNA-induced gene silencing, while MoDCL1 rarely contributes to siRNA production from hairpin RNA (Kadotani et al., 2004; Kadotani et al., 2008). *M. oryzae* sRNAs have diverse genomic origins and dynamically changed according to the appressorium development, diverse stresses and during rice infection (Nunes et al., 2011; Raman et al., 2013). Profiling of sRNAs from RNAi mutants showed that MoDCL2, MoRdRP2, and MoAGO3 are responsible for sRNA production and transcript regulation, particularly from transposons and intergenic regions (Raman et al., 2017). Sequencing of Ago-binding sRNA libraries suggested that MoAGO3 is the major AGO protein in RNA silencing of transposons and viral RNAs (Nguyen et al., 2018). However, non-canonical sRNAs have not been studied in *M. oryzae* or in other plant pathogenic fungi.

In this study, we comparatively profiled canonical and non-canonical sRNAs by sequencing sRNA libraries from the wild-type (WT), DCLs mutants. We showed that Dicer-dependent sRNAs share features with canonical sRNAs, whereas Dicer-independent sRNAs have distinct characteristics, suggesting non-canonical RNAi mechanisms in *M. oryzae*. We also identified a non-canonical RNAi component, MoERI-1. MoERI-1-dependent sRNAs may be involved in vegetative growth and conidiation of *M. oryzae*.

## Materials and methods

### Generation of targeted-gene deletion mutant and complementation

*M. oryzae* KJ201 (wild type) was obtained from the Center for Fungal Genetic Resources (CFGR) at Seoul National University, Seoul, Korea. To produce targeted gene deletion construct, the upstream and downstream flanking regions of RNAi-associated genes (*MoDCL1*, *MoDCL2*, and *MoERI*-1) were amplified from the genomic DNA (gDNA) of the wild type. The hygromycin B phosphotransferase gene (HPH) cassette and the geneticin resistance cassette were amplified from pBCATPH and pII99, respectively (Lee et al., 2003; Kim et al., 2005). Constructs for targeted-gene deletion were produced by double-joint PCR using upstream flanking, downstream flanking, and hygromycin/geneticin-resistance cassette (Supplementary Table 4). Transformation and selection of mutants were performed as previously described (Lim and Lee, 2020). To generate the double gene deletion mutant of *MoDCL1* and *MoDCL2*, MoDCL2 was replaced with HPH construct first, and the geneticin-resistance construct were introduced into Δ*Modcl2* protoplasts. For complementation of *MoERI-1*, constructs containing ORF and promoter were amplified from the gDNA of the wild type and inserted into Δ*Moeri-1* protoplasts with geneticin cassette. The complemented strain was selected on TB3 agar with 800 ppm of geneticin, and selected by PCR using the ORF primers. All strains used in this research were deposited in the CFGR^1^.

### Extraction and sequencing of sRNA and mRNA

To prepare total mycelial RNA, fungal mycelia were incubated in liquid complete medium (CM) at 25°C for 4 days and collected for total mycelial RNA extraction. For preparation of total RNA from conidiation stage, conidiating mycelia were harvested as previously described (Park et al., 2013). Briefly, fungal mycelia incubated in CM at 25°C for 4 days were collected and placed on sterile membrane filter (Whatman, Maidstone, England) laid on V8 agar medium. After 6 days of incubation sealed with parafilm, followed by 1 day unsealed incubation for aeration, the membrane filter with conidiation stage were collected and subjected to RNA extraction. Total RNA including sRNA was isolated using miRNeasy mini kit (Qiagen, Hilden, Germany) according to manufacturer’s instructions. The quality and concentration of each sample were checked by Agilent 2100 Bioanalyzer or 2200 TapeStation (Agilent Technologies, CA, USA). Three mycelia samples of each strain were independently used for RNA extraction, and the RNA samples were pooled for the synthesis of one cDNA library. Three independent cDNA libraries for the WT and two for mutant strains were constructed (KJL1-3, D1L1-2, D2L1-2, and D12L1-2). Construction of cDNA libraries of sRNA samples and their sequencing were performed at Macrogen (Seoul, Korea). cDNA libraries of sRNAs were constructed from total RNA samples using TruSeq Small RNA Library Prep Kit (Illumina, CA, USA) and sequenced by Illumina Hiseq2500. cDNA library construction and sequencing of mRNA were performed at National Instrumentation Center for Environmental Management at Seoul National University (NICEM, Seoul, Korea). cDNA libraries were constructed using TruSeq RNA Sample Prep Kit (Illumina) and paired-end sequencing of each sample was conducted by Illumina HiSeq2500.

### sRNA and mRNA data analysis

As the first step of raw sRNA read processing, adaptor sequences were removed by Cutadapt v1.8.1 (Martin, 2011). Reads were filtered by quality and size using Sickle v1.33 (Joshi and Fass, 2011). Reads ranging 18–30-nt with at least two copies in each library were used for genome mapping to reduce the possible sequencing error and degradation products. Processed reads were mapped to the reference genome of the *M. oryzae* strain 70–15 (MG8) from National Center for Biotechnology Information (NCBI)^2^ using bowtie v1.2.2 (Langmead et al., 2009). Read abundance was counted by the number of copies and multiple-mapped reads were weighted by ShortStack3 with “U” method (Johnson et al., 2016). Distinct genome-matched reads were calculated based on the hits. To compare the different libraries by fixing the coordinate, sRNA loci were clustered by ShortStack3. The abundance of each loci was normalized to reads per kilobase per million reads (RPKM). sRNA loci with following conditions were used for further analyses: distinct read > 5, combined read abundance of loci > 10 RPKM, length of loci > 100 bp sRNA loci ≥ 4-fold differences in mutant libraries compared to the WT libraries were referred to as “Dicer or MoERI-1 dependent loci” in this study. Dicer-independent sRNA loci were determined as sRNA loci excluding the loci with more than twofold decrease in mutant libraries compared to the WT libraries.

Raw mRNA reads were processed to remove low-quality reads and trim adapter sequences using NGS QC Toolkit v2.3.3 (Patel and Jain, 2012). The resulting reads were mapped against the *M. oryzae* reference genome (MG8) using HISAT2 v2.0.4 (Kim et al., 2019). The transcriptome was assembled using the genome-guided method of StringTie v1.3.3 (Pertea et al., 2015). We used fragments per kilobase of transcript per million mapped read pairs (FPKM) as the expression value.

### Mycelial growth, conidiation, conidial germination, and appressorium formation

All strains were cultured in modified complete agar medium (CMA) and minimal agar medium (MMA) at 25°C for 9 days for assessment of mycelial growth and colony morphology. Conidia were collected from cultures on V8 agar after incubation for 7 or 11 days. For aeration, plates were unsealed for 24 h on the last day of incubation. Conidiation was measured under a microscope using a hemacytometer. To assess conidial germination and appressorium formation, 50 μL of conidial suspensions (2 × 10^4^/mL) was dropped on a hydrophobic cover glass at 25°C. Conidial germination and appressorium formation were evaluated under a microscope after incubation for 2 and 8 h, respectively. Conidiogenesis was observed as described previously (Lau and Hamer, 1998; Goh et al., 2011). Hyphae from conidia were incubated with Calcofluor white (CFW, 10 μg/mL, Sigma Aldrich, USA) at 25°C for 5 min to stain septa, and visualized using a fluorescence microscope (Carl Zeiss, Oberkochen, Germany).

### Pathogenicity test

Pathogenicity tests were performed using the susceptible rice cultivar, Nakdongbyeo (*Oryzae sativa*) as previously described (Lim et al., 2018). Briefly, to perform spray inoculation assay, 10 mL of conidia suspension (5 × 10^4^/mL, supplemented with 250 ppm of Tween 20) was inoculated on 4-week-old rice seedlings. Inoculated rice plants were incubated in humid and dark chamber at 25°C for 1 day (100% relative humidity) and then incubated in growth chamber at 28°C for 5 days. For performing sheath inoculation assay, conidia suspension (2 × 10^4^/mL) was inoculated into the sheath of 6-week-old of rice. Inoculated rice sheath was incubated at 25°C for 48 h in a humid chamber. After then, invasive hyphal growth was observed under a microscope.

## Results

### Profiling of canonical sRNAs in *M. oryzae*

**FIGURE 1:**
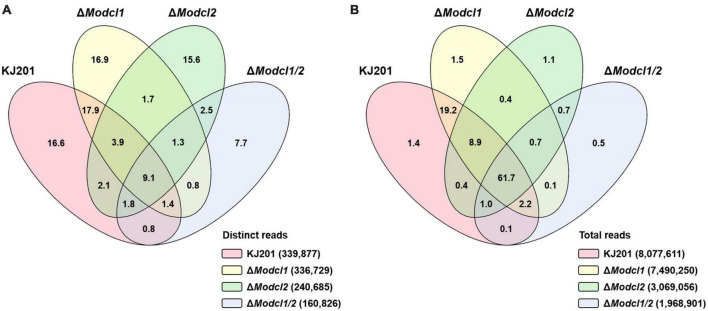
Venn diagram of sRNAs profiled in the WT and *MoDCL* mutant libraries. Venn diagram showing the proportions of **(A)** distinct reads and **(B)** total reads in the four libraries. Numbers in each section are percentages of read numbers relative to the sum of distinct reads or total reads from the four libraries.

**FIGURE 2:**
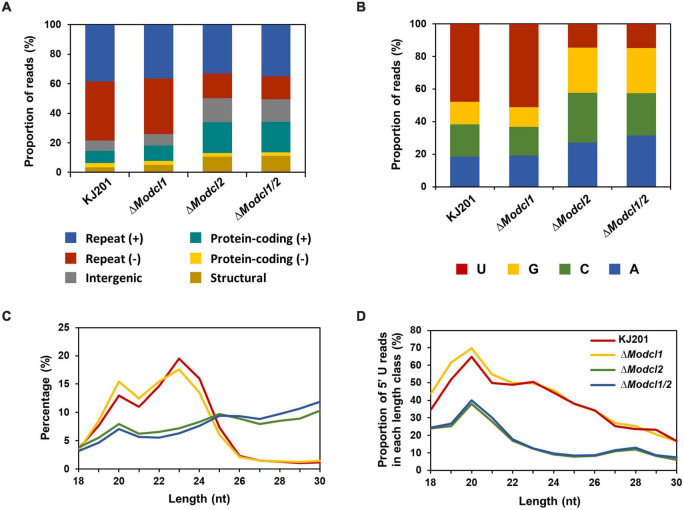
Characteristics of *M. oryzae* sRNAs. **(A)** Genomic origins of sRNAs. (+) and (–), sense strand and antisense strands, respectively. **(B)** Nucleotide composition at the 5′-ends of sRNAs. **(C)** Length distribution of sRNAs. **(D)** Proportions of 5′-uracil reads in each length class.

To profile *M. oryzae* sRNAs in the canonical RNAi pathway, two single deletion mutants (Δ*Modcl1* and Δ*Modcl2*) and one double deletion mutant (Δ*Modcl1/2*) were generated (Supplementary Figure 1). From mycelia of the WT strain KJ201 and the deletion mutants, size-fractionated small RNAs were isolated and used for cDNA library construction and sequencing (Supplementary Table 1). Sequencing of two KJ201 libraries (KJL1 and KJL2) revealed discrete patterns, mostly due to altered sRNA lengths (Supplementary Figures 2A,B). Although the length distribution of KJL1 reads peaked at 20-nt, KJL2 showed a major peak at 23-nt and minor peak at 20-nt. To assess repeatability, we performed additional experiment of KJ201 sequencing. The sequencing result of KJL3 was similar to that of KJL2 (Supplementary Figures 2A,B). Distinct reads shared by replicates accounted for > 89% of the total reads, implying that the different length distribution of KJL1 was caused by a change in the abundance of the common distinct reads (Supplementary Figures 2D,E). Pearson correlation analysis based on the read abundance of sRNA-producing loci showed that the read abundances of sRNA-producing loci between KJ201 replicates are highly correlated (Supplementary Figure 2C). Although the majority of sRNA-producing loci were maintained through replicates, we selected the sequencing results of the library set in second experiments (KJL2, D1L2, D2L2, and D12L2) for further analyses. After adaptor removal and quality filtering, reads of 18–30-nt were mapped to the *M. oryzae* reference genome, resulting in 2–8 million genome mapped reads. By comparing sRNA reads among the four libraries, reads in KJ201 and Δ*Modcl1*, but not Δ*Modcl2* and Δ*Modcl1/2*, which are highly likely to be generated by MoDCL2, accounted for the largest portion (17.9%) of whole distinct reads, and the second-largest portion (19.2%) of whole total reads (Figures 1A,B). Reads detected in all four libraries might include the both types of reads produced in Dicer-dependent and -independent manner. Such common reads accounted for only 9.1% of distinct reads, but the largest portion (61.7%) of total reads. We next investigated the genomic origin, length distribution and 5′-end frequencies of the sRNAs. The mapped reads from *M. oryzae* WT mainly originated from the repeat region (79.4%) in which there were almost equal amounts of sRNAs from sense and antisense strands (Figure 2A). The nucleotides at the 5′-ends were highly biased toward uracil (47.9%) and most reads were 19–24-nt in length (88% of total reads), with a peak at 23-nt (Figures 2B,C). The genomic origin proportions, length distributions and 5′-end frequencies of Δ*Modcl1* were similar to those of the WT. Comparison of KJ201 and Δ*Modcl1* showed that the common reads accounted for 94.9% of total reads and mainly affected the sRNA features of each library (Supplementary Figures 4A–C). In *MoDCL2*-deleted mutants (Δ*Modcl2* and Δ*Modcl1/2*), the proportions of sRNAs from the antisense strand of repeats were decreased, and those from the sense strands of exons were increased compared to the WT (Figure 2A). In addition, those biases toward certain size classes (19–24-nt) and 5′-end uracil in the wild type were abolished by the deletion of *MoDCL2* (Figures 2B,C). In a comparison of reads against KJ201, ∼20% of distinct reads and ∼70% of total reads were shared (Supplementary Figure 5A). This proportion of common reads between KJ201 and Δ*Modcl2* was smaller than that between KJ201 and Δ*Modcl1* (Supplementary Figures 4A, 5A). Unlike the *MoDCL2*-deleted mutant libraries, the KJ201 library-specific reads accounted for large proportions of both distinct reads and total reads possibly because of the Dicer-dependent sRNAs. Common reads between KJ201 and *MoDCL2* mutant libraries were mainly derived from the repeat region with sense orientation (Supplementary Figures 5B,C). Intriguingly, the proportions of 5′-U reads of each size peaked at 19–20-nt not only in the WT and Δ*Modcl1* but also in Δ*Modcl2* and Δ*Modcl1/2* (Figure 2D and Supplementary Figure 3C).

### Identification of sRNA-producing loci

**FIGURE 3:**
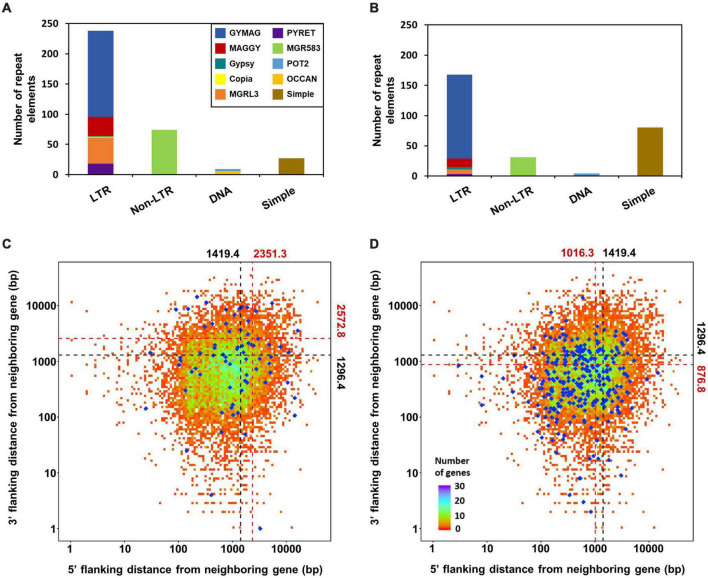
Relationship between sRNA loci and repeat elements. Repeat elements associated with **(A)** Dicer-dependent and **(B)** Dicer-independent sRNA loci. Nearest-neighbor gene distance density plot for genes associated with **(C)** Dicer-dependent, and **(D)** Dicer-independent sRNA loci. Total protein-coding genes are plotted as the background; blue diamonds, genes associated with sRNA loci. Average distances are shown above and on the right, and are plotted as dotted lines. Black and red, total protein-coding genes and genes with sRNA loci, respectively.

**FIGURE 4:**
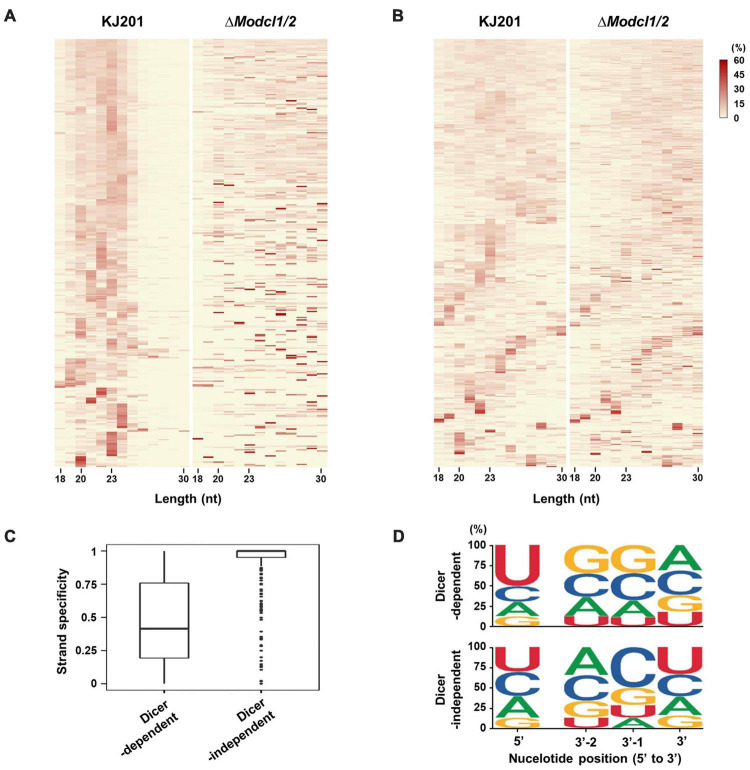
sRNA features according to Dicer dependency. Size distributions of sRNAs from **(A)** Dicer-dependent sRNA loci and **(B)** Dicer-independent sRNA loci as heatmaps. **(C)** Strand-specificity of sRNAs from sRNA-producing loci. **(D)** Probability of nucleotide composition at the 5′-end position and three positions from the 3′-end, using sRNAs from Dicer-dependent and -independent sRNA loci. Nucleotide composition analysis at whole positions is shown in Supplementary Figure 5.

sRNA-producing loci were clustered with genome-aligned reads by ShortStack3. Among 6,974 clustered loci, 939 were determined as sRNA-producing loci by following criteria: length ≥ 100-nt, number of distinct reads ≥ 5, and sum of read number from a locus ≥ 10 reads per kilobase per million (RPKM). Dicer-dependent sRNA-producing loci were defined as sRNA-producing loci with fourfold decrease in normalized reads in Dicer-deleted mutants compared to the WT. Deletion of *MoDCL1* and *MoDCL2*, and double deletion of Dicer-coding genes decreased sRNA reads abundance in 94, 354, and 335 loci, respectively (Supplementary Figure 6). Most of MoDCL1-dependent sRNA-producing loci overlapped with genes (95%; 89 out of 94), compared to 18 and 21% of MoDCL2- and MoDCL1/2-dependent sRNA loci, respectively (Table 1). Only 22 MoDCL1-dependent sRNA loci were overlapped with repeat elements, 20 of which were simple repeats. Additionally, 87 and 84% of MoDCL2- and MoDCL1/2-dependent sRNA loci overlapped with repeat elements, and most sRNA loci-associated repeat elements were LTR-retrotransposons. In a comparative analysis of canonical and non-canonical sRNAs, 335 loci that showed fourfold decrease in Δ*Modcl1/2* were selected as Dicer-dependent sRNA loci, and 438 loci that showed no significant decrease in Δ*Modcl1/2* were determined as Dicer-independent sRNA loci (Table 1). Among Dicer-independent sRNA loci, most (67%) of them were associated with genes, followed by transposons, simple repeats and intergenic regions. Among Dicer-dependent sRNA loci-related repeat elements, an LTR-retrotransposon, GYMAG was the most frequent (41%), followed by the non-LTR type, MGR 583 (21%) (Figure 3A). By contrast, GYMAG accounted for 40% of repeat elements associated with Dicer-independent sRNA loci, followed by simple repeats (21%) and MGR583 (9%) (Figure 3B). More than half of the gene-associated Dicer-dependent loci were also associated with repeat elements. Therefore, we hypothesized that genes associated with Dicer-dependent loci were located at genome regions with low gene density. As expected, genes associated with Dicer-dependent loci were more distant from neighboring genes than the average of protein-coding genes (Figure 3C). In the theory of two speed genome evolution, repeat rich regions are gene sparse, resulting much longer flanking distance between two genes (Dong et al., 2015). However, genes associated with Dicer-independent sRNA loci showed shorter-than-average flanking distances (Figure 3D). sRNAs from Dicer-dependent sRNA-producing loci were predominantly 19–24-nt range in WT and were irregularly distributed in 18–30-nt when *MoDCL1/2* were deleted (Figure 4A). However, sRNAs from Dicer-independent sRNA loci showed erratic length distribution patterns with or without Dicer proteins (Figure 4B). Compared to Dicer-dependent sRNA loci, Dicer-independent loci showed higher strand-specificity, indicating a greater likelihood of generating sRNAs from given strand (Figure 4C). sRNAs from Dicer-dependent loci showed uracil preference at the 5′ end mainly due to 19–24-nt sRNAs, whereas sRNAs from Dicer-independent loci presented biased nucleotide composition with cytosine at the penultimate position, which was mainly observed in 25–30-nt sRNAs (Figure 4D and Supplementary Figure 7).

**TABLE 1:** Number of sRNA-producing loci according to Dicer-dependency.

Dependency	Total	Gene	Intergenic	LTR-retrostransposon	Non-LTR retrotransposon	DNA transposon	Simple repeat
MoDCL1	94	89	8	2	0	0	20
MoDCL2	354	64	16	208	88	8	16
MoDCL1/2	335	72	15	190	73	9	18
Dicer-independent	438	294	89	134	28	4	65
MoERI-1	109	99	10	1	0	0	21

### Identification of MoERI-1, a non-canonical RNAi component

In non-canonical RNAi pathways, the central roles of Dicer and Ago are substituted by other exonucleases and Ago-like proteins. To identify the substitutes of the core RNAi component, Dicer, in non-canonical RNAi pathway, BLAST analyses of the previously reported non-canonical RNAses were performed. A well-conserved exonuclease, ERI-1, which was reported in *N. crassa*, showed high homology (100% coverage, 83.7% identity) with the protein coded by *MGG_07327*, therefore, *MGG_07327* was designated as *MoERI-1* (Supplementary Figure 8). ERI-1 of *N. crassa* is responsible for the production of disiRNA, a non-canonical sRNA class of 22-nt major length and a 5′-end uracil bias, similar to QDE-2-binding sRNAs (Lee et al., 2010; Dang et al., 2016). Based on the high homology between two proteins, we hypothesized that MoERI-1 might have roles in Dicer-independent sRNA pathway in *M. oryzae*.

### Deletion of MoERI-1 reduced growth rate, increased conidiation, and caused abnormal septum formation

**FIGURE 5:**
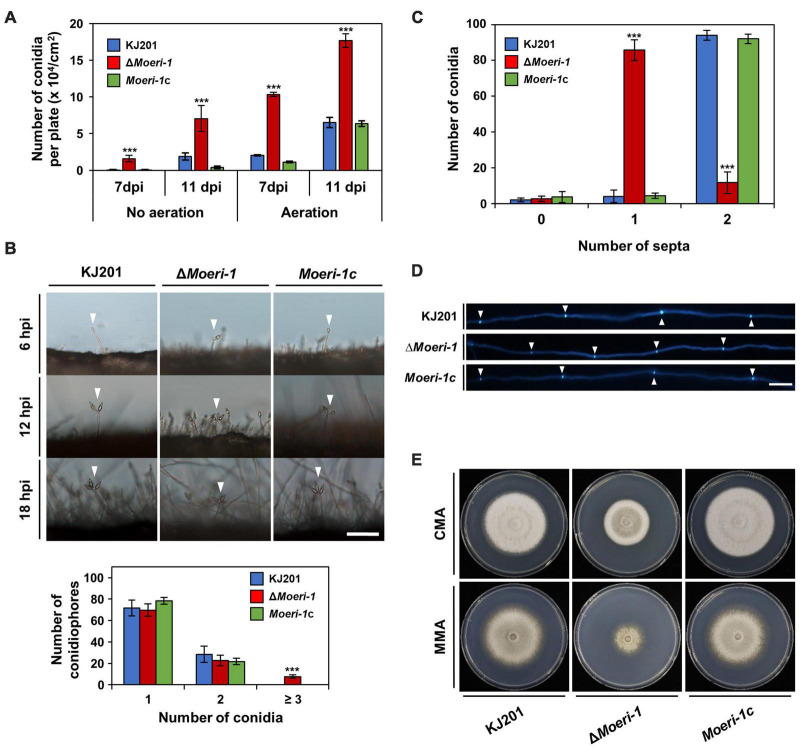
Phenotypes of Δ*Moeri-1*. **(A)** Conidiation assay of WT, Δ*Moeri-1*, and the *MoERI-1* complemented strain, *Moeri-1*c, with or without aeration. Conidia of each strain were collected at 7 and 11 days post-inoculation (dpi). Each strain was counted in triplicate. **(B)** Conidiophores were observed under light microscopy at 6, 12, and 18 h post-induction (hpi). White arrowheads indicate the conidiophores with the conidia. One hundred conidiophores of each sample were counted at 12 hpi in triplicate. Scale bar, 40 μm. **(C)** The number of conidia according to septum number was counted. One hundred conidia of each sample were counted in triplicate. Error bars indicate the standard deviations of mean values. ***p < 0.001, Student’s *t*-test between the mean values of the WT and the mutant strains. **(D)** Calcofluor white staining of mycelial septa. Scale bar, 40 μm. **(E)** Mycelial growth on CMA and MMA at 25°C 9 days post inoculation.

**FIGURE 6:**
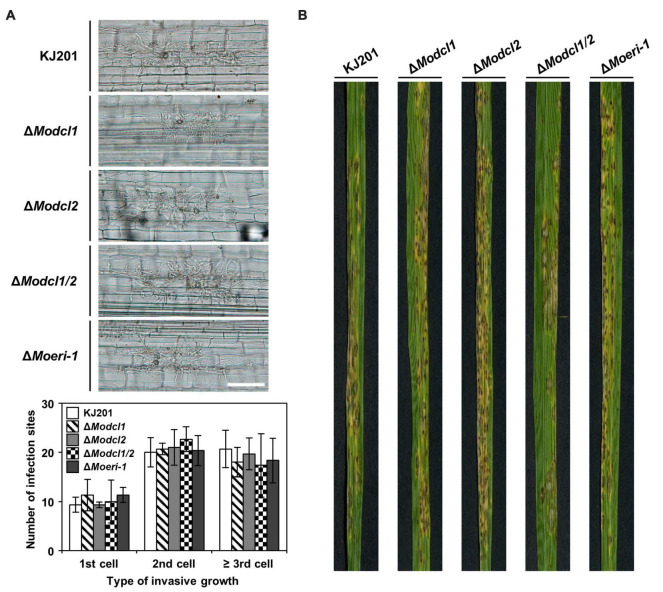
Pathogenicity of *Magnaporthe oryzae* WT and deletion mutants. **(A)** Conidial suspensions (2 × 10^4^/mL) were inoculated onto rice sheath cells of 6-week-old Nakdong. Invasive growth of the WT and deletion mutants in sheath cells at 48 h post-inoculation was observed under light microscopy. The types of invasive growth were rated as 1st cell, 2nd cell, or ≥ 3rd cell colonization. Fifty infection sites of each sample were counted in triplicate. Error bars indicate the standard deviations of mean values. Student’s *t*-test between the mean values of the WT and the mutant strains indicated that there are no significant differences. **(B)** 4-week-old cv. Nakdong rice seedlings were sprayed with conidial suspensions (5 × 10^4^/mL) of the WT and deletion mutant strains. Disease severity was measured at 7 days post-inoculation.

We generated a gene deletion mutant by the homologous recombination (Supplementary Figure 1) and performed phenotypic analyses. Irrespective of aeration and incubation duration, Δ*Moeri-1* showed increased conidiation compared to the WT (Figure 5A). Additionally, the conidiophores carrying first conidia were observed earlier in Δ*Moeri-1* than the WT and Δ*Moeri-1* conidiophores had more conidia than the WT at the same time points (Figure 5B). Deletion of *MoERI-1* decreased the size of conidia and increased the proportion of conidia with one septum (Figure 5C). Such abnormal septum formation also observed in mycelia of Δ*Moeri-1*, with shorter distance between neighboring two septa (Figure 5D). Δ*Moeri-1* also showed decreased mycelial growth rate compared to the WT (Figure 5E). Altered phenotypes were restored in the complemented strain, *Moeri-1c* (Figures 5A–E and Supplementary Figure 9). Conidial germination, appressorium formation, and pathogenicity of Δ*Moeri-1* were not changed like the Dicer-deleted mutants (Figure 6 and Supplementary Table 3).

### Transcriptome analysis of Δ*Moeri-1*

**FIGURE 7:**
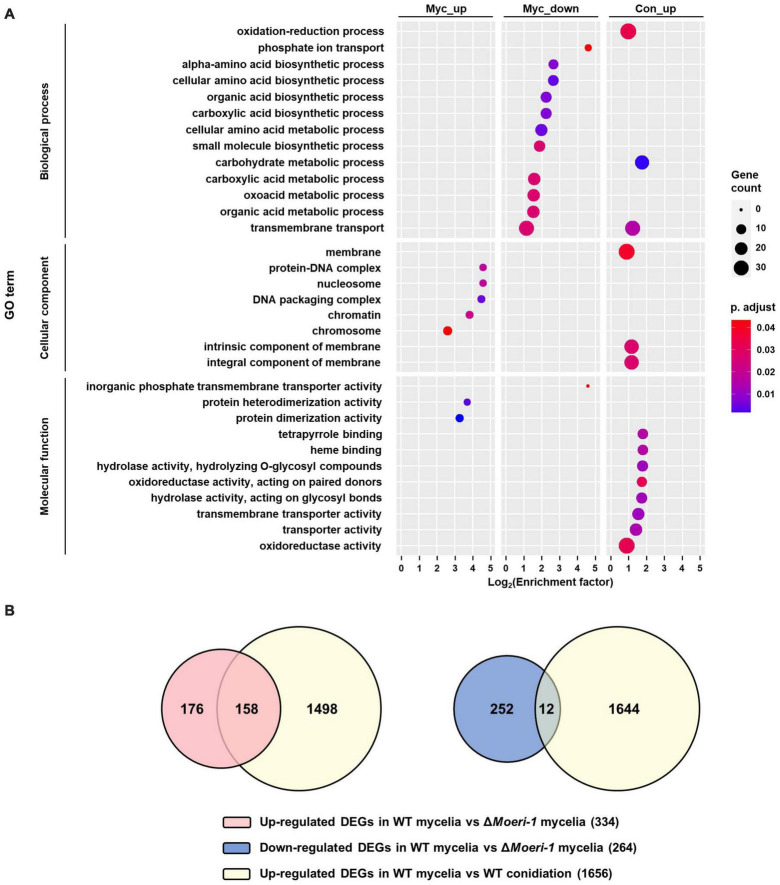
DEG analyses between WT and Δ*Moeri-1*. **(A)** GO enrichment analysis was performed for DEGs in mycelia of WT and Δ*Moeri-1*, and during conidiation in WT with BLAST2GO (Conesa and Götz, 2008). Circle size denotes the number of enriched genes; circle color denotes the adjusted *p*-value; *x*-axis, enrichment factor. **(B)** Venn diagram between the DEGs by *MoERI-1* deletion in mycelia and the up-DEGs during conidiation in WT.

For further elucidation of the molecular mechanisms underlying altered phenotypes of Δ*Moeri-1*, we performed mRNA sequencing using total RNAs isolated from the mycelia and conidiation stage in the WT and in Δ*Moeri-1*. Deletion of *MoERI-1* altered the expression of 598 genes in mycelia and 811 genes in the conidiation stage. Gene Ontology (GO) enrichment analysis yielded 19 and 14 enriched GO terms in the DEGs of the mycelia and conidiation stages, respectively (Figure 7). Among the down-regulated DEGs of the mycelia, 11 terms were enriched in biological processes (BP), 9 terms for “metabolic process” (e.g., “alpha-amino acid biosynthetic process”; GO: 1901607) and two for “transport” (“transmembrane transport”; GO: 0055085, and “phosphate ion transport”; GO: 0006817). One term, “inorganic phosphate transmembrane transporter activity” (GO: 0005315), was enriched in molecular function (MF). Among DEGs upregulated at the mycelia stage, no term was enriched in BP, two “protein binding”-related terms were enriched in MF (“protein dimerization activity”; GO: 0046983, and “protein heterodimerization activity”; GO: 0046982) and five terms related to “protein-containing complex” and “chromosome” (e.g. “nucleosome”; GO: 0000786, and “chromatin”; GO: 0000785) were enriched in cellular component (CC). Among downregulated DEGs at the conidiation stage, two “metabolic process” terms (“carbohydrate metabolic process”; GO: 0005975, and “oxidation-reduction process”; GO: 0055114), and one term, “transmembrane transport” (GO: 0055085) were enriched in BP. For the MF category, four “catalytic activity” terms, two “binding” and two “transporter activity” terms were enriched (e.g., “hydrolase activity, hydrolyzing O-glycosyl compounds”: GO: 0004553, “heme binding”: GO: 0020037 and “transmembrane transporter activity”; GO: 0022857). Three terms enriched in CC were related to the term “membrane” (e.g., “integral component of membrane”; GO: 0016021).

We hypothesized that the direct or indirect effect of derepression of conidiation-related genes might affect the phenotypes of Δ*Moeri-1*. Therefore, we investigated overlaps between DEGs resulting from conidiation in the WT and *MoERI-1* mutants at the mycelia and conidiation stages. Of the genes upregulated by *MoERI-1* deletion at the mycelia stage, 48% (160 of 334) overlapped with those up-regulated in the WT due to conidiation (Figure 7B). On the contrary, only 8% (21 of 264) of the genes downregulated by the deletion of *MoERI-1* at the mycelial stage overlapped with those downregulated due to conidiation in the WT. Therefore, the up-regulated DEGs caused by deletion of *MoERI-1* in mycelia considerably overlapped with those up-regulated DEGs resulting from conidiation of the WT. Among the up-regulated DEGs were five genes (MAP kinase gene *MCK1*, mitophagy-related gene *MoATG24*, C2H2 zinc finger transcription factor *CONx6*, chitinase-coding gene *MoChia1*, and anti-apoptosis protein-coding gene *MoTCTP*), whose deletion mutants showed reduced conidiation (Jeon et al., 2008; He et al., 2013; Lilin et al., 2013; Cao et al., 2016; Yang et al., 2019). However, their expression was not significantly different at the conidiation stage between Δ*Moeri-1* and the WT. We also found genes encoding proteins important in cell cycle control such as *MoPCL1* and a homolog of kinetochore protein-encoding gene *MIS14* among the overlapped up-regulated genes (Holt and May, 1996; Lilin et al., 2013).

### Profiling of sRNAs in Δ*Moeri-1*

Based on the phylogenetic, phenotypic, and transcriptome data, we hypothesized that MoERI-1 regulates mRNA levels of other genes during the mycelia and conidiation stages. sRNA of the Δ*Moeri-1* from mycelia and conidiation stages were subjected to deep sequencing (Supplementary Table 2). Deletion of *MoERI-1* decreased the proportions of repeat-derived sRNAs (from 50 to 35%) and increased the read proportions of intergenic regions and structural RNAs (from 4 to 22% and 21 to 27%, respectively) at the conidiation stage (Supplementary Figure 10). By contrast, 5′-end nucleotide proportions and length distributions were little affected by the deletion of *MoERI-1*. To investigate the relationship between Dicer proteins and MoERI-1, we compared MoERI-1-dependent sRNAs with Dicer-dependent or –independent sRNAs. 70% of the MoERI-1-dependent sRNAs were overlapped with Dicer-dependent sRNAs, and most of the overlapped total sRNAs were derived from repeat elements (81%) (Supplementary Figures 11A,B). These Dicer-dependent, MoERI-1-dependent sRNAs also shared the other features of Dicer-dependent sRNAs like 5′ U preference and 23-nt length bias (Supplementary Figures 11B,C). Meanwhile, Dicer-independent, MoERI-1-dependent sRNAs were mainly derived from tRNAs, and showed relatively irregular patterns in length distributions and nucleotide compositions (Supplementary Figures 12B,C).

In a sRNA clustering analysis, 109 sRNA-producing loci showed a fourfold decrease when *MoERI-1* was deleted. Among 109 MoERI-1-dependent sRNA loci, 50 loci were Dicer-independent but lacked specific sRNA features. Among the 109 loci, 22 were associated with repeat elements and 99 with 99 protein-coding genes (Table 1). Among the 99 protein-coding genes, the RNA seq data showed that six genes, including *MoChia*, were upregulated in Δ*Moeri-1.*

## Discussion

sRNAs generally have specific patterns such as length or nucleotide bias at a specific position, which were reported in both canonical and non-canonical RNAi (Eamens et al., 2008; Lee et al., 2010; Nicolas et al., 2010). In this study, most *M. oryzae* sRNAs are 19–24-nt in length (Figure 2C). The deletion of *MoDCL2* led to disruption of the most length bias, while the deletion of *MoDCL1* caused a minor decrease of 23–24-nt sRNAs. Previously, sRNA sizes made by DCLs were experimentally speculated (Kadotani et al., 2008). The size distribution of siRNAs produced by *MoDCL1*-overexpressed mutant is included in the size distribution of siRNAs made by MoDCL2, but is concentrated within a relatively narrow range of longer sRNAs. However, with the normal level of expression, MoDCL1 was unable to make enough siRNAs to induce RNAi (Kadotani et al., 2008). Combined with our results, MoDCL2 is the major Dicer protein responsible for the biogenesis of 19-24-nt sRNAs, while MoDCL1 has a minor role in the production of 23–24-nt sRNAs during the mycelial stage.

Size distribution of *M. oryzae* sRNAs showed 23- and 20-nt as highest and second peaks, respectively (Figure 2C). It has been revealed that sRNAs of different lengths are involved in the discrete RNAi pathway in other eukaryotes (Hamilton et al., 2002). Two distinct classes of sRNAs (25–26- and 21-nt) have also been reported in an oomycete pathogen, *Phytophthora parasitica*, suggesting that the 25–26-nt class mediates RNAi. The 21-nt sRNAs, another major class, are involved with highly transcribed genomic loci in the WT (Jia et al., 2017). However, in *M. oryzae*, 20-nt sRNA loci and 23-nt sRNA loci are highly correlated, indicating that most 20- and 23-nt sRNAs are derived from the same loci. Immature longer sRNAs undergo additional size processing after loading into AGO proteins in many organisms (Xue et al., 2012; Marasovic et al., 2013). AGO proteins of *M. oryzae* bind to 20-nt siRNAs with uracil at the 5′-end, but do not participate in sRNA biogenesis (Raman et al., 2017; Nguyen et al., 2018). Considering the features of functional sRNAs, 23-nt sRNAs of *M. oryzae* may be processed into 20-nt class during the RNAi pathway.

We found that one of the WT libraries, KJL1 presented a distinct sRNA length distribution against other replicate libraries (KJL2 and KJL3) (Supplementary Figures 2A,B). However, the sRNA-producing loci between replicates were highly correlated (Supplementary Figure 2C). This result indicates that the sRNA loci repertoire was similar between replicates, but the length profiles of sRNAs derived from those loci were changed. In *M. oryzae*, diverse length distribution patterns have been reported not only according to various endogenous and environmental changes but also through the control WT libraries (Nunes et al., 2011; Raman et al., 2013; Raman et al., 2017; Nguyen et al., 2018). Combined with our results, these findings suggest that the length distribution of total sRNAs in *M. oryzae* is changed dynamically by apparent factors, including stress, tissue, and strain, and by unknown minor factors.

ex-siRNA (*F. graminearum* and *M. circinelloides*) and milRNA (*N. crassa* and *Penicillium marneffei*) are involved in the regulation of fungal gene expression (Lee et al., 2010; Nicolas et al., 2010; Lau et al., 2013). In *M. oryzae*, most Dicer-dependent sRNA loci within the genes were concentrated on the exon (Figure 4A and Supplementary Figure 5B). sRNAs derived from the genes also showed lower strand-specificity than those of Dicer-independent sRNAs, indicating *M. oryzae* sRNAs from genes were ex-siRNAs (Figure 4C). To find milRNA of *M. oryzae*, we performed miRNA loci prediction using software miREAP. However, we could not find miRNA loci with significant read numbers. A previous study found *milR236* by comparing sRNA libraries prepared from appressorium samples of *M. oryzae* strains. Overexpression of *milR236* caused suppression of *MoHat1*, leading to delayed appressorium formation (Li et al., 2020). We found that *milR236* was in the sRNA library of KJ201 WT, and its abundance was reduced in Δ*Modcl1/2* compared to the WT library. However, the sRNA-producing locus containing *milR236* was removed at the loci-filtering step because of the small read number. These findings imply that the lack of functional sRNAs in the KJ201 mycelial library might result from stage-specific expressions and roles of functional sRNAs, including *milR236*.

Deletion of *MoDCL* genes disrupted biased sRNA patterns. However, Dicer-independent sRNAs still had significant preferences on 20-nt with 5′U (Figure 2D). Moreover, Dicer-independent sRNAs showed cytosine bias at the penultimate position (Figure 4D). These specific patterns indicate a non-canonical, Dicer-independent RNAi pathway in *M. oryzae*. Similar to cytosine bias at the penultimate position in *M. oryzae*, rdrp-dependent degraded RNAs (rdRNAs) of *M. circinelloides* were reported to prefer uracil at the penultimate position (Trieu et al., 2015). RdRP and a non-canonical ribonuclease, R3B2 were necessary for the biogenesis and penultimate nucleotide bias of rdRNAs (Trieu et al., 2015). MoRdRP2 was reported to be involved in the canonical RNAi pathway, but the relevance between MoRdRP-dependent sRNA-producing loci and Dicer-independent sRNAs were not shown (Raman et al., 2017). Additionally, we could not find the homolog of *R3B2* in *M. oryzae*. This implies that there should be an unknown Dicer-independent RNAi pathway exploiting penultimate-cytosine sRNAs. In *N. crassa*, sequencing of Ago-bound sRNA revealed a non-canonical sRNA, disiRNA with a 22-nt length bias and preference for uracil at the 5′-end (Lee et al., 2010). A homolog of *NcERI-1*, *MoERI-1* was identified, and associated sRNAs were profiled. Δ*Moeri-1* showed increased conidiation and reduced mycelial growth (Figure 5). Furthermore, comparison of mRNA and sRNA seq data revealed that MoERI-1-dependent sRNAs might be involved in gene regulation. However, unlike disiRNA of *N. crassa*, MoERI-1-dependent sRNAs showed an irregular pattern.

By comparative profiling, we found discrete features of sRNAs generated in Dicer-dependent and Dicer-independent manner. Based on the patterns of sRNAs in Δ*Modcl1/2*, we suggest the possibility of non-canonical RNAi pathways in *M. oryzae*. Furthermore, we identified a non-canonical RNAi component, *MoERI-1*, and revealed that MoERI-1 has roles in conidiation and septum formation of *M. oryzae*. Our study suggests the importance of non-canonical RNAi in the study of plant pathogenic fungi and may provide a novel roadmap for a comprehensive understanding of both canonical and non-canonical RNAi in fungi and beyond.

## Data availability statement

The data presented in this study are deposited in the National Center for Biotechnology Information Sequence Read Archive under the BioProject ID PRJNA856435.

## Author contributions

HL and Y-HL conceived and designed the study, discussed and interpreted the results, and contributed to the writing of the manuscript. HL and Y-JL carried out experiments. HL and GC analyzed the data. All authors have read and approved the final manuscript.

## Footnotes

1
http://genebank.snu.ac.kr

2
https://www.ncbi.nlm.nih.gov/

## Supplementary material

Supplementary Data Sheet 1 DEGs in mycelia stage.

Supplementary Data Sheet 2 DEGs in conidiation stage.

Supplementary Data Sheet 3 sRNA producing loci.

Supplementary Data Sheet 4 Supplementary Tables.

Supplementary Data Sheet 5 Supplementary Figures.
