# Supplementary material for: Comparative profiling of canonical and non-canonical small RNAs in the rice blast fungus, Magnaporthe oryzae
Source: Front Microbiol. 2022 Sep 26;13:995334. doi: 10.3389/fmicb.2022.995334 (PMC9549407; doi:10.3389/fmicb.2022.995334)
Supplement: Supplementary Data Sheet 4 — Supplementary Tables. [file Data_Sheet_4.pdf]

Supplementary Table 1. Statistical summary of mycelial sRNA sequencing and processing

	KJ201		Δ*Modcl1*		Δ*Modcl2*		Δ*Modcl1/2*		Δ*Moeri-1*	
	Redundant	Distinct	Redundant	Distinct	Redundant	Distinct	Redundant	Distinct	Redundant	Distinct
Size & quality filtered-reads	25,513,775	2,373,285	26,651,112	2,427,881	21,819,728	1,462,440	15,456,677	1,116,322	17,908,050	1,855,090
Reads ≥ 2	24,001,969	861,479	25,074,785	851,554	20,851,803	494,515	14,692,959	352,604	16,736,831	683,871
Genome-mapped reads	8,077,611	339,877	7,490,250	336,729	3,069,056	240,685	1,968,901	160,826	5,959,877	280,201
Nonstructural reads	7,550,911	325,225	6,834,360	321,466	2,266,710	223,004	1,426,058	146,435	5,636,951	268,493

Supplementary Table 2. Statistical summary of sRNA sequencing of vegetative mycelia (myc) and conidiating mycelia (con)

	**KJ201-myc**		**KJ201-con**		**Δ*Moeri-1*-myc**		**Δ*Moeri-1*-con**	
	Redundant	Distinct	Redundant	Distinct	Redundant	Distinct	Redundant	Distinct
Size & quality filtered-reads	43,927,137	2,631,573	38,061,678	1,153,789	33,263,172	2,386,238	34,940,019	1,095,591
Reads ≥ 2	42,504,595	1,209,031	37,320,907	413,018	31,864,903	987,969	34,219,981	375,553
Genome-mapped reads	17,358,180	446,597	4,217,598	135,392	12,480,183	395,684	4,888,736	133,151
Nonstructural reads	16,653,800	432,358	2,861,727	125,225	11,849,967	381,761	3,101,024	120,910

Supplementary Table 3. Conidial germination and appressorium formation of the strains

Strain	Germination*	Appressorium formation*
KJ201	92.3 ± 0.6[a,b**]	98.6 ± 1.5[a]
Δ*Modcl1*	93.7 ± 0.6[a]	96.6 ± 3[a]
Δ*Modcl2*	89.3 ± 3.1[b]	98.6 ± 1.2[a]
Δ*Modcl1/2*	94.3 ± 1.5[a]	98.6 ± 1.2[a]
Δ*Moeri-1*	91 ± 2[a,b]	98.6 ± 1.5[a]
*Moeri-1*C	94 ± 3[a]	99.3 ± 0.6[a]

*Values are means ± SD of three replications (n = 100).

**Values in the same column with the same superscript letter are not statistically different using Duncan's Multiple Range Test ($P < 0.05$).

Supplementary Table 4. Primers used in this study

Name	Primers
MoDCL1_5'F	ACTGGTCGCGCATACTCCTC
MoDCL1_L+5'R	cct cca cta gct cca gcc aag ccGCG TAC TTG GCA CAT TTC CTG C
MoDCL1_L+3'F	gtt ggt gtc gat gtc agc tcc gga gGGCTAGGGTAGAGCCCATATACAC
MoDCL1_3'R	CCA CGC AGA TGT GAA GAT GAC CTC
MoDCL1_5'NF	CTCCGAGACCTGCTCTTTAGTGC
MoDCL1_3'NR	GTC CTT GAT CCC CAC ATG GTC C
MoDCL2_5'F	GAGATGAATGGCGACTCCATCGAG
MoDCL2_L+5'R	cct cca cta gct cca gcc aag ccGGT AGC AGA GTG GGC GGT AAT G
MoDCL2_L+3'F	gtt ggt gtc gat gtc agc tcc gga gAAG GAG AAA CCG CGG CCT AC
MoDCL2_3'R	CAC TGA TGA CAG GAC TCC CTG AG
MoDCL2_5'NF	TCGCACAGGGAGGTTAGCTTG
MoDCL2_3'NR	CAA GGG TAT CTG CTG CCG TCT G
MoERI-1_5'F	AAAGCAACCTCACACCGCATGTC
MoERI-1_L+5'R	cct cca cta gct cca gcc aag ccGGC AAG AAG CCG AAC AGT GGA
MoERI-1_L+3'F	gtt ggt gtc gat gtc agc tcc gga gAAG TCG CCC GCG TAA GAT CAA CA
MoERI-1_3'R	TGC TCA TGA CGC TCC AGG TCA TAC
MoERI-1_5'NF	CCA CGC GAA GAT GTC AGT GTC A
MoERI-1_3'NR	CGG GGA TCA GTG ACG AAC AAC TCA

*Letters in lower case indicate HPH linker sequence.
