# Supplementary material for: Comparative profiling of canonical and non-canonical small RNAs in the rice blast fungus, Magnaporthe oryzae
Source: Front Microbiol. 2022 Sep 26;13:995334. doi: 10.3389/fmicb.2022.995334 (PMC9549407; doi:10.3389/fmicb.2022.995334)
Supplement: Supplementary Data Sheet 5 — Supplementary Figures. [file Data_Sheet_5.pdf]

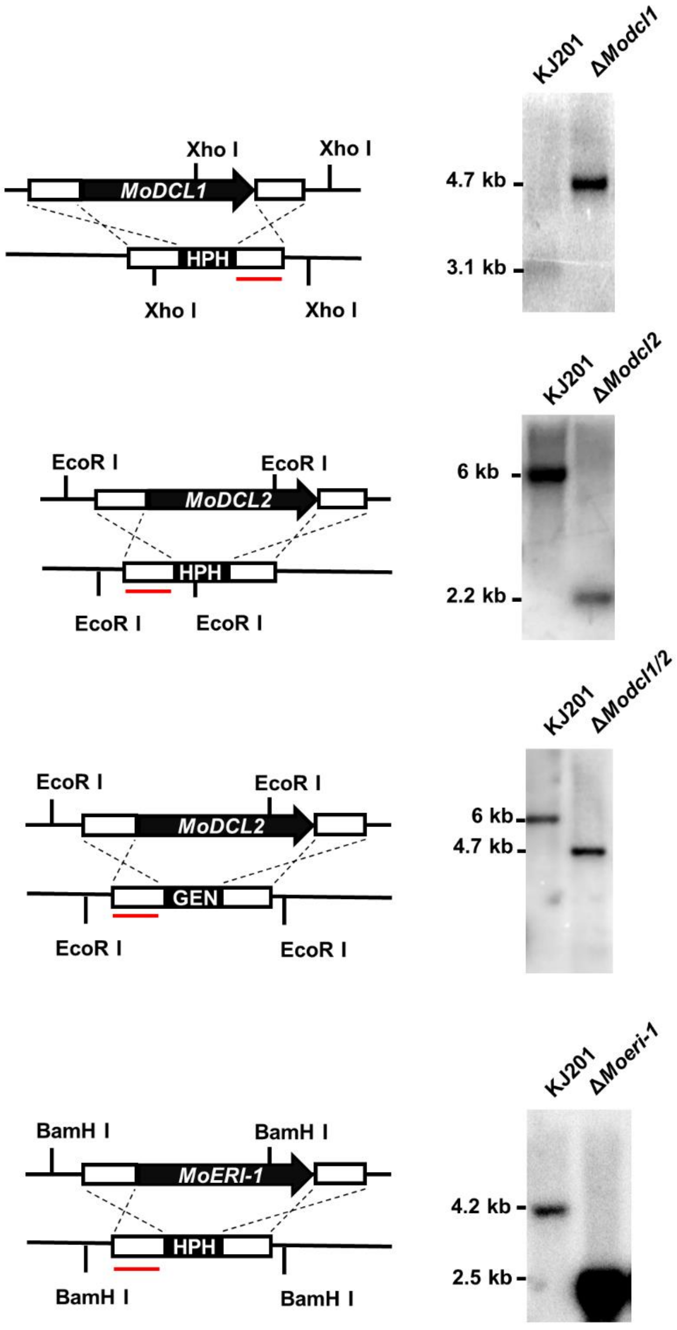

Supplementary Figure 1. Confirmation of target gene deletion by Southern blotting. Genomic DNAs of KJ201 (WT) and deletion mutants were isolated and digested with Xho I, EcoR I, or BamH I. The upstream or downstream construct of each gene was used as a probe for Southern blot analysis. Red bar indicates the location of the probe used in each experiment. Primers used in the experiments are listed in Supplementary Table 4.

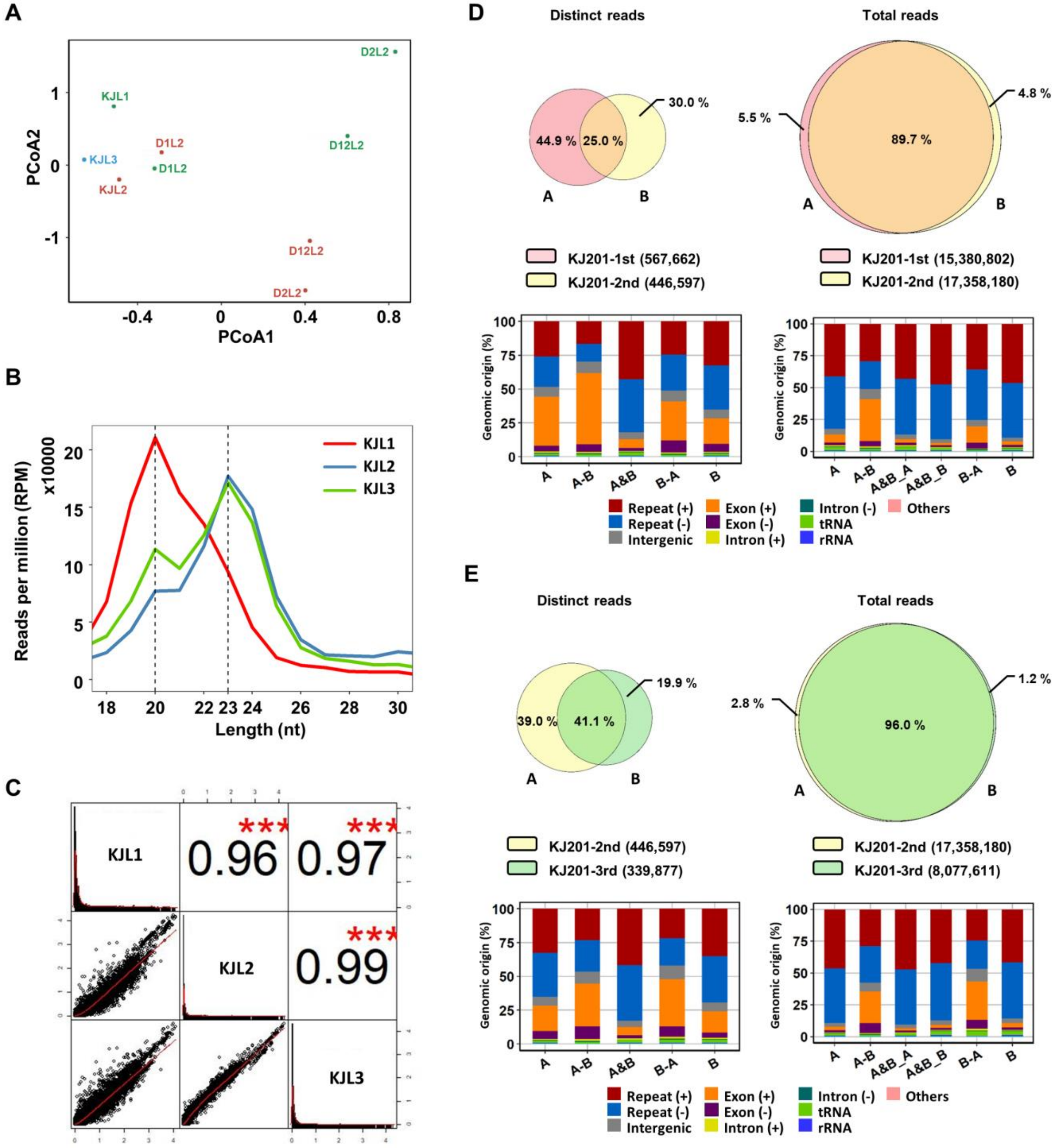

Supplementary Figure 2. Repeatability of sRNA seq data. (A) Principal coordinates (PCoA) plot of sRNA seq libraries. **(B)** Length distributions of the KJ201 WT sRNA seq libraries. **(C)** Pearson correlation coefficients between the WT libraries based on sRNA loci. *** $p < 0.001$. **(D)**Venn diagram of reads of KJ201-first and KJ201-second among distinct and total reads. **(E)** Venn diagram of reads of KJ201-second and KJ201-third among distinct and total reads. **(D and E)** Genomic origins are shown in the lower panel. “A” and “B” indicate the two libraries in the Venn diagram, “A-B” and “B-A’ are the differences between two sets, and “A&B” indicates the intersection of two libraries. Because the distinct reads of “A&B” can have different total read numbers in each library,

the intersection was divided into “A&B_A” and “A&B_B” to compare the genomic origins of total reads.

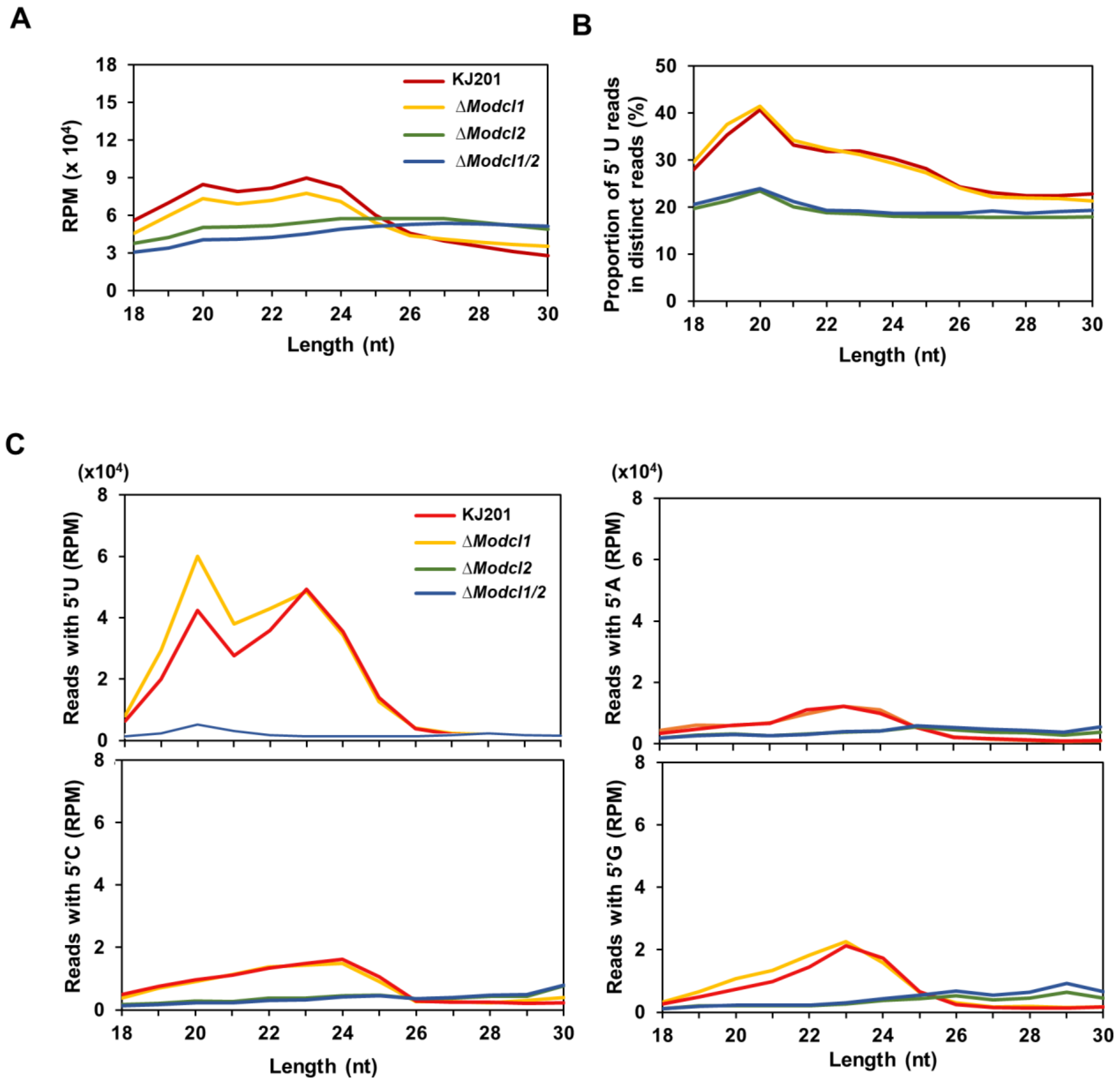

Supplementary Figure 3. Length distributions of sRNAs. (A) Length distribution of distinct reads. **(B)** Proportions of reads with uracil at the 5'-end among the distinct reads. **(C)** Distribution of total reads according to 5'-end nucleotide.

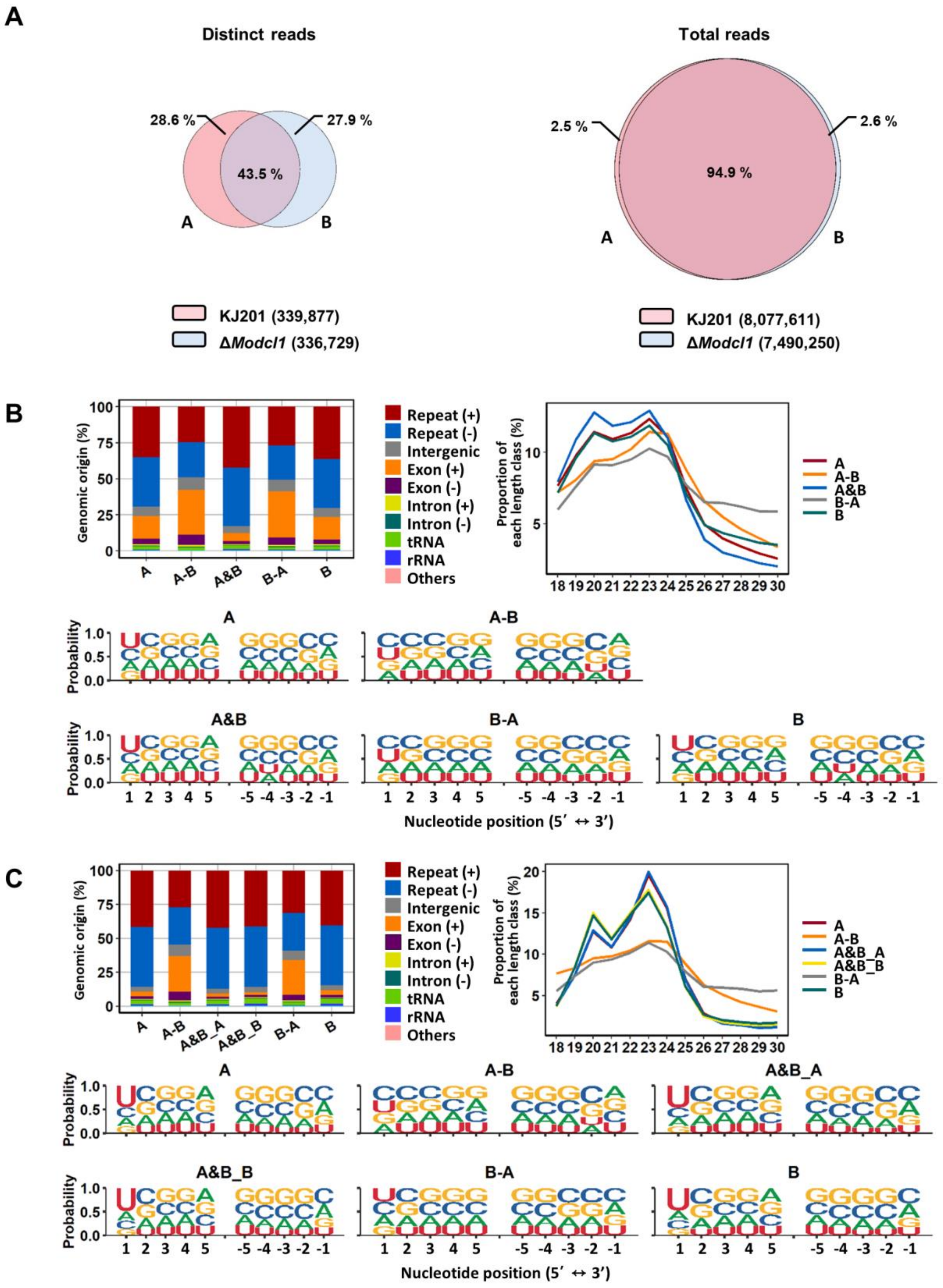

Supplementary Figure 4. Venn diagram of KJ201 and Δ*Modcl1* libraries using distinct and total sRNAs. (A) Venn diagram showing the proportions of distinct and total reads from KJ201 and Δ*Modcl1*. Genomic origin, length distribution, and nucleotide composition were investigated using the distinct reads **(B)** and total reads **(C).**

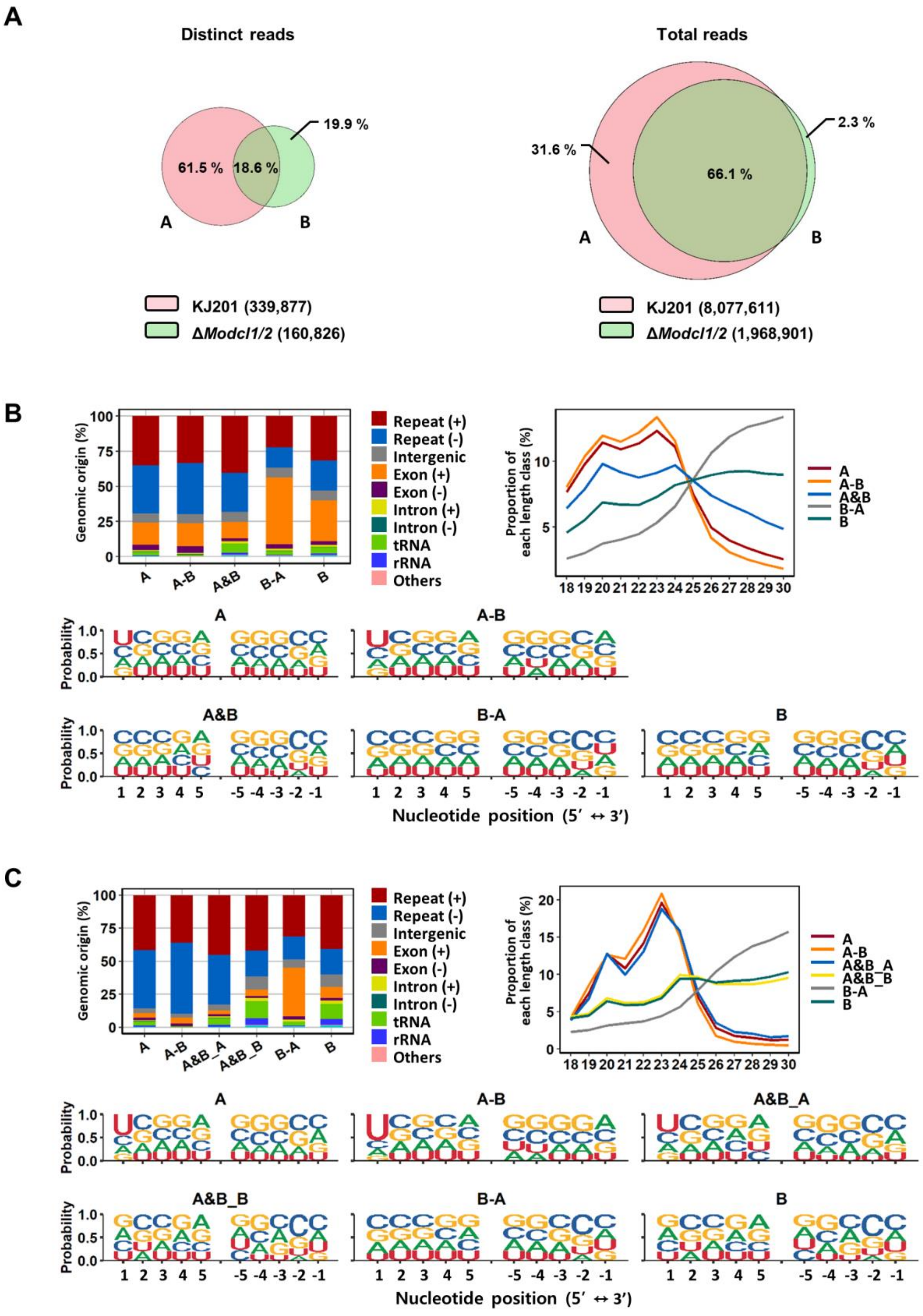

Supplementary Figure 5. Venn diagram of KJ201 and Δ*Modcl1/2* libraries using distinct and total sRNAs. (A) Venn diagram of the proportions of distinct and total reads from KJ201 and

Δ*Modcl1/2*. Genomic origin, length distribution, and nucleotide composition were investigated using the distinct reads **(B)** and total reads **(C)**.

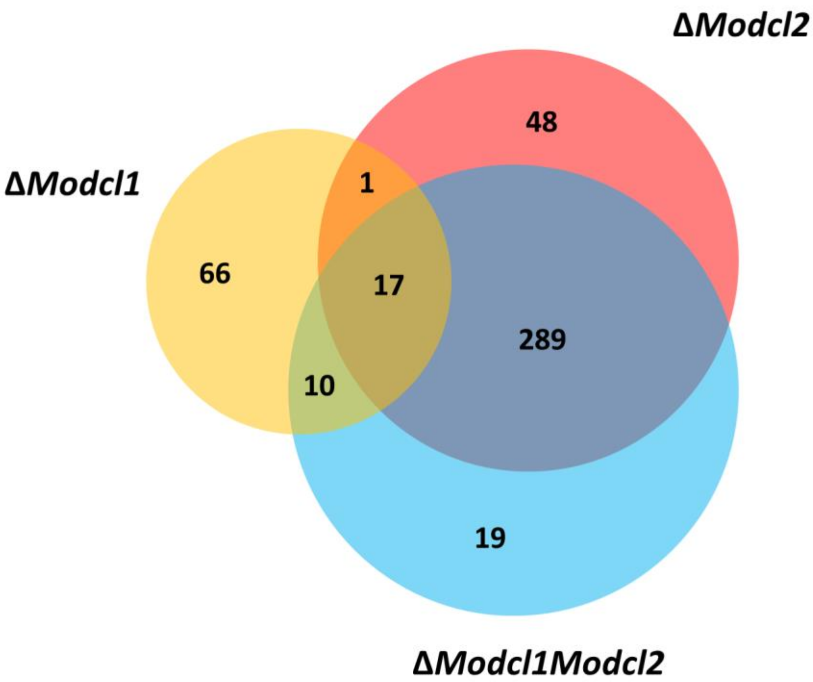

Supplementary Figure 6. Venn diagram of sRNA loci with reduced sRNAs in the dicer mutants.

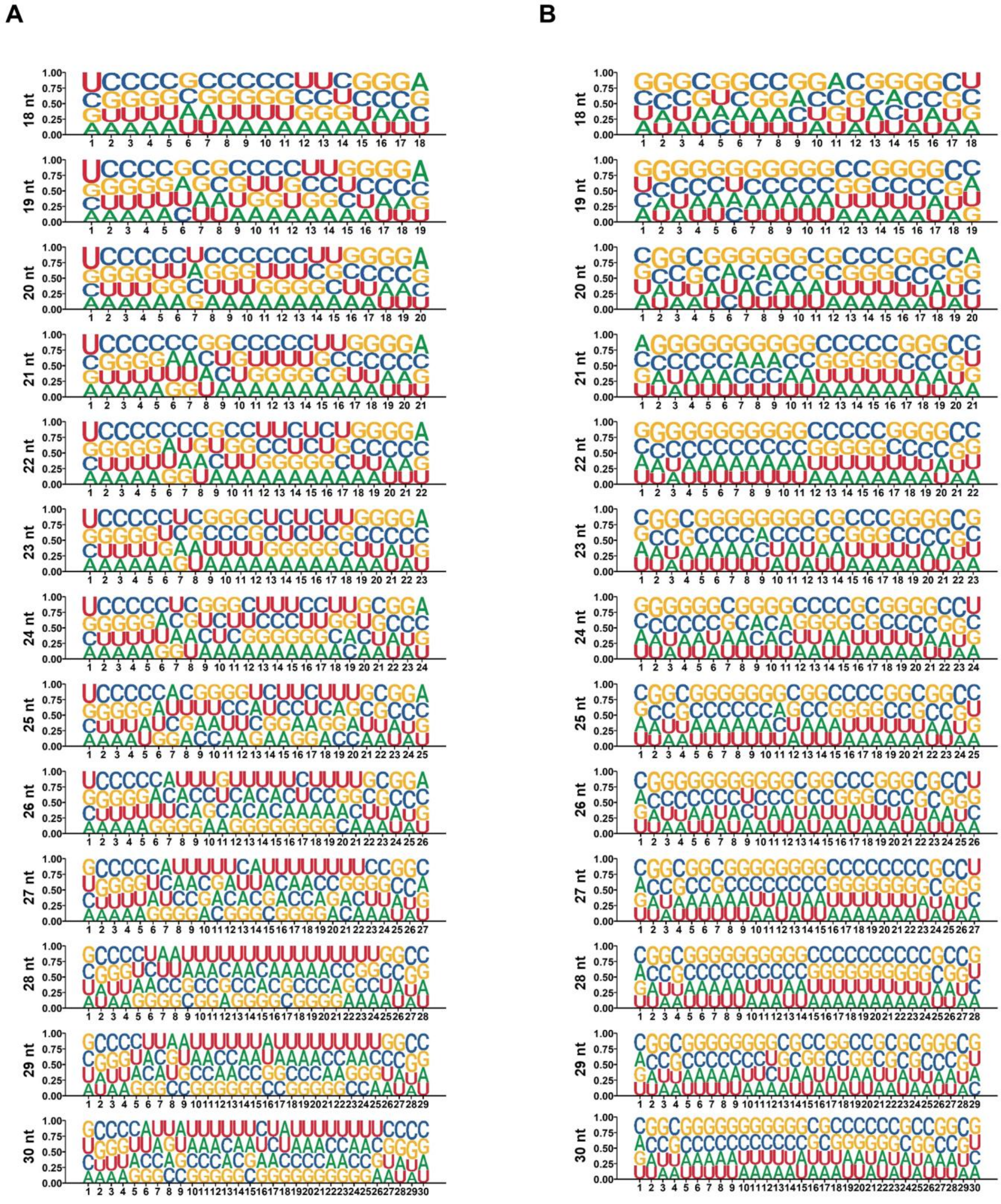

Supplementary Figure 7. Nucleotide compositions according to positions. sRNAs from **(A)** Dicer-dependent and **(B)** Dicer-independent sRNA loci. *x*-Axis, nucleotide positions on sRNAs from 5’ to 3’.

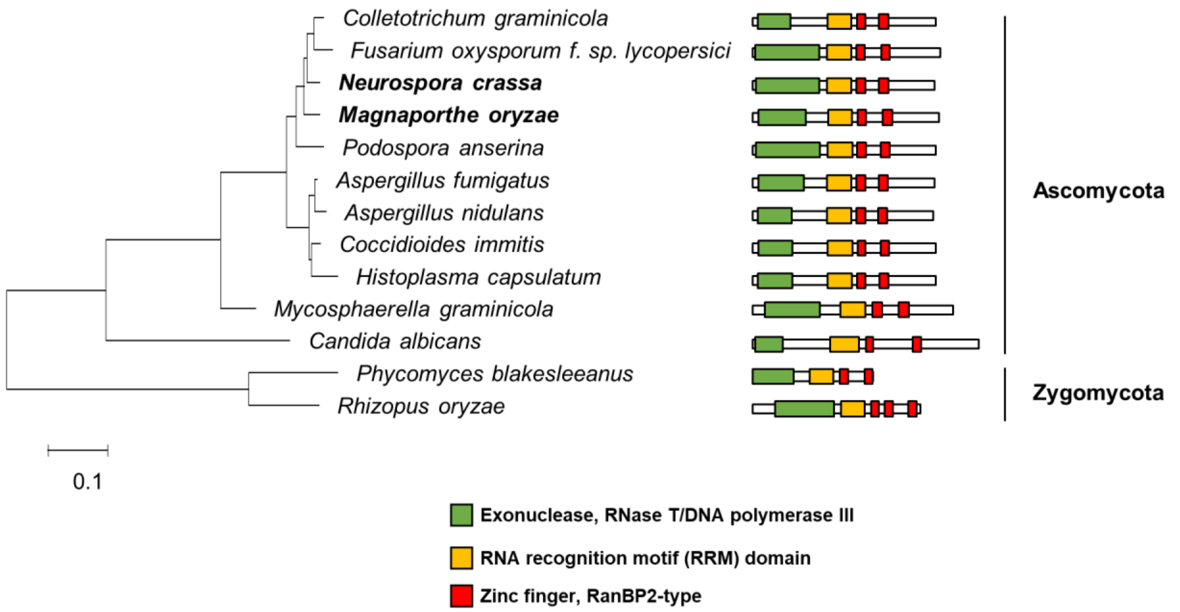

Supplementary Figure 8. Phylogenetic analysis of fungal ERI-1 proteins. Neighbor-joining was performed by alignment of the exonuclease, RRM, and zinc-finger domains of *ERI-1* homologues confirmed by BLAST MASTRIX (CFGP; http://cfgp.riceblast.snu.ac.kr) using Goldstandard 3.0.

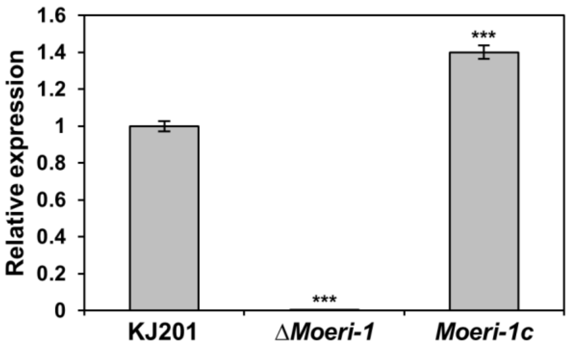

Supplementary Figure 9. Expression of *MoERI-1*. Expression was measured in the WT, Δ*Moeri-1*, and the complemented strain *Moeri-1c* by qRT-PCR. β-tublin was used as a reference gene. Error bars indicate the standard deviations of mean values. ***p < 0.001, Student's t-test between the mean values of the WT and the mutant strains.

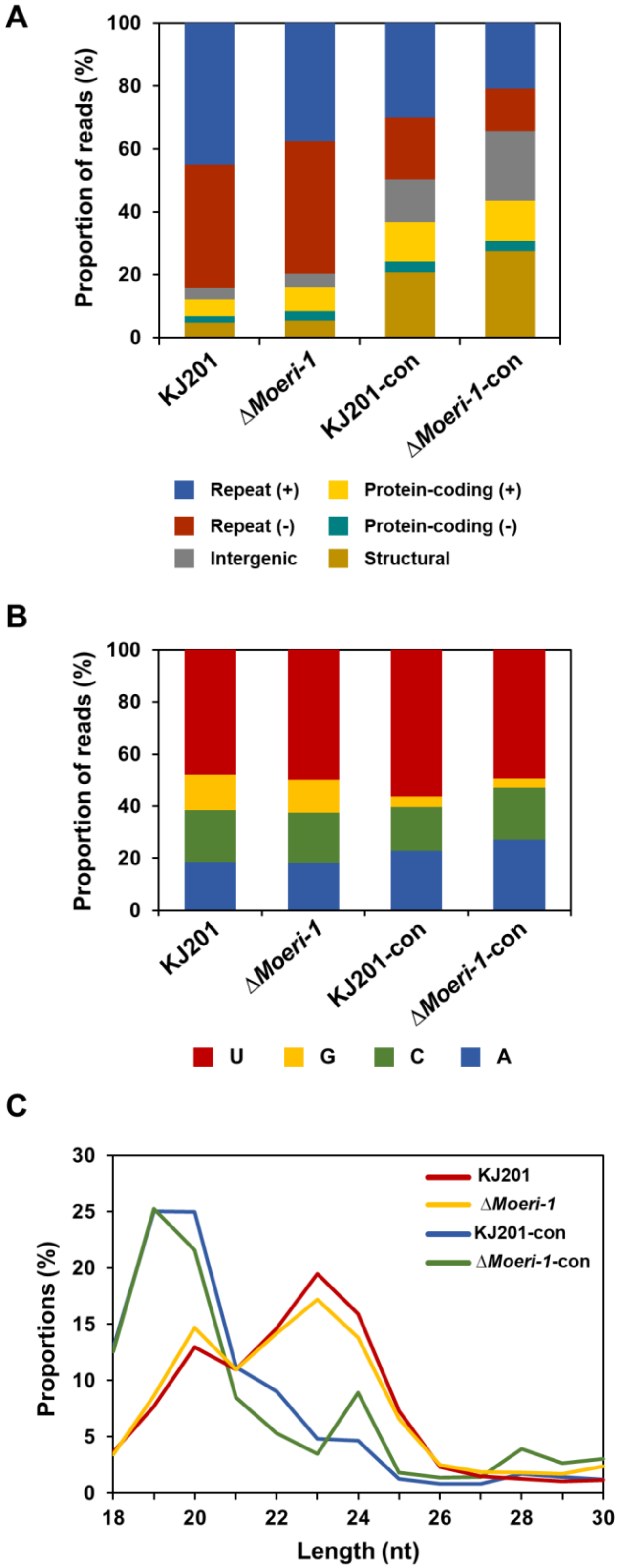
A
100
80
60
40
20
0
Proportion of reads (%)
KJ201
ΔMoeri-1
KJ201-con
ΔMoeri-1-con
Repeat (+)
Repeat (-)
Intergenic
Protein-coding (+)
Protein-coding (-)
Structural
B
Proportion of reads (%)
KJ201
ΔMoeri-1
KJ201-con
ΔMoeri-1-con
U
G
C
A
C
30
25
20
15
10
5
0
Proportions (%)
18
20
22
24
26
28
30
Length (nt)
KJ201
ΔMoeri-1
KJ201-con
ΔMoeri-1-con

Supplementary Figure 10. Profiling of sRNAs in the WT and Δ*Moeri-1* in the mycelia and during conidiation. (A) Genomic origins of sRNAs. **(B)** 5'-end nucleotide preference. **(C)** Length distribution.

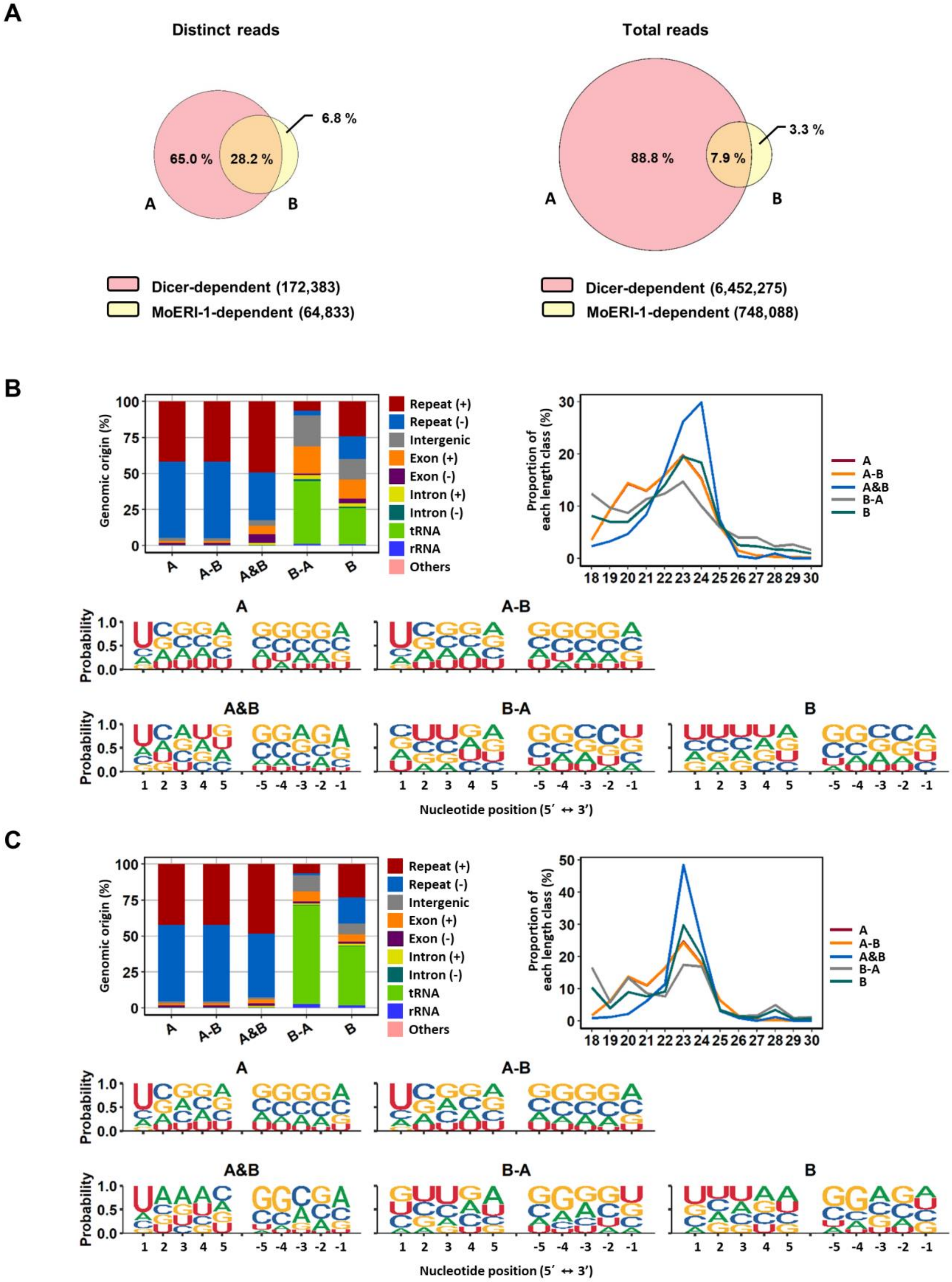
A
Distinct reads
65.0 %
28.2 %
6.8 %
A
B
Dicer-dependent (172,383)
MoERI-1-dependent (64,833)
Total reads
88.8 %
7.9 %
3.3 %
A
B
Dicer-dependent (6,452,275)
MoERI-1-dependent (748,088)
B
Genomic origin (%)
A
A-B
A&B
B-A
B
Repeat (+)
Repeat (-)
Intergenic
Exon (+)
Exon (-)
Intron (+)
Intron (-)
tRNA
rRNA
Others
Proportion of each length class (%)
18 19 20 21 22 23 24 25 26 27 28 29 30
A
A-B
A&B
B-A
B
Probability
Nucleotide position (5′ ↔ 3′)
C
Genomic origin (%)
Repeat (+)
Repeat (-)
Intergenic
Exon (+)
Exon (-)
Intron (+)
Intron (-)
tRNA
rRNA
Others
Proportion of each length class (%)
A
A-B
A&B
B-A
B
Probability
Nucleotide position (5′ ↔ 3′)

Supplementary Figure 11. Venn diagram of Dicer-dependent and MoERI-1-dependent reads using distinct and total sRNAs. (A) Venn diagram of the proportions of distinct and total reads in the WT using Dicer-dependent and MoERI-1-dependent reads. Genomic origin, length distribution, and nucleotide composition were investigated using the distinct **(B)** and total **(C).**

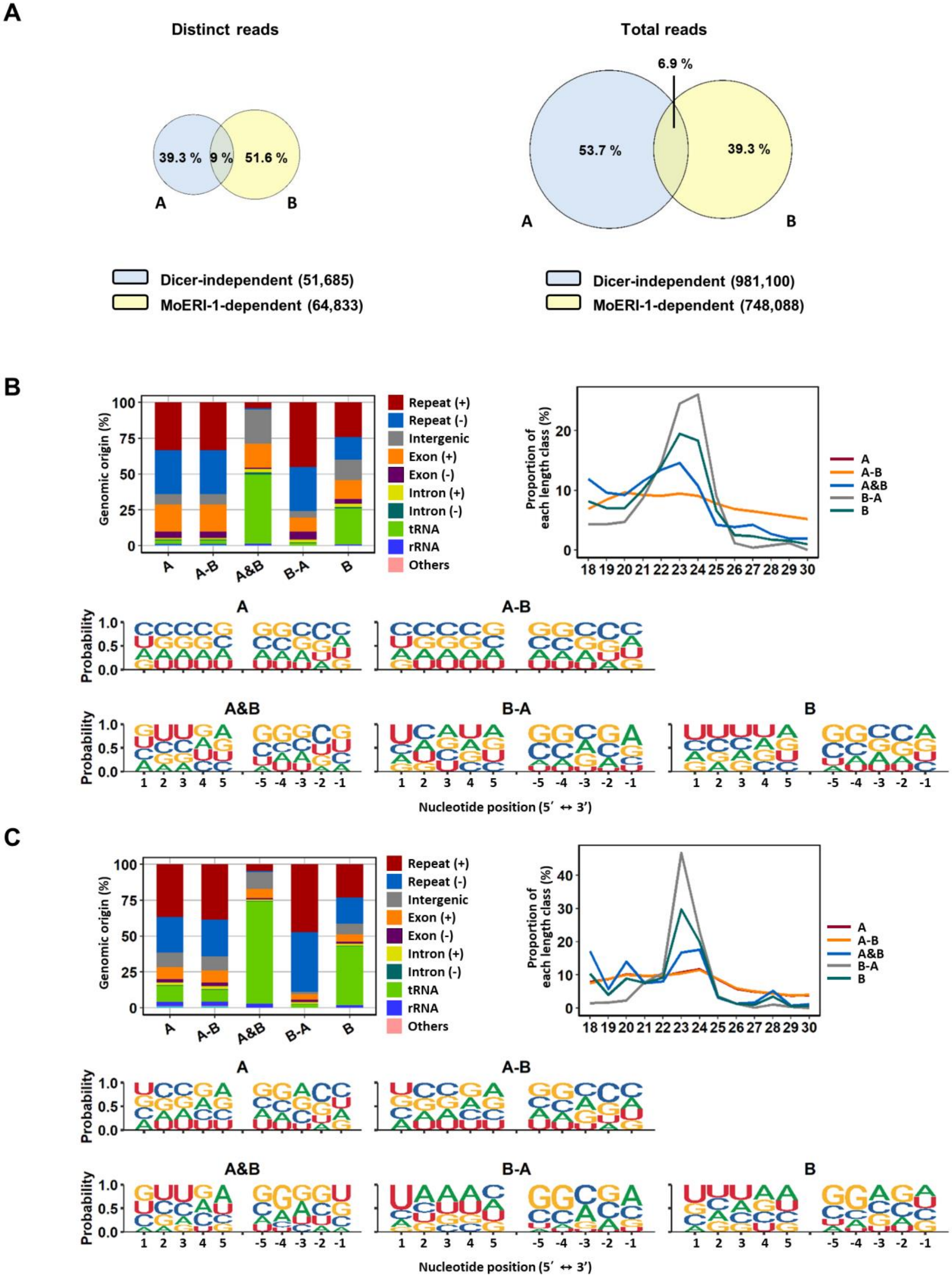
A
Distinct reads
39.3 %
9 %
51.6 %
A
B
Total reads
6.9 %
53.7 %
39.3 %
A
B
Dicer-independent (51,685)
MoERI-1-dependent (64,833)
Dicer-independent (981,100)
MoERI-1-dependent (748,088)
B
Genomic origin (%)
100
75
50
25
0
A
A-B
A&B
B-A
B
Repeat (+)
Repeat (-)
Intergenic
Exon (+)
Exon (-)
Intron (+)
Intron (-)
tRNA
rRNA
Others
Proportion of each length class (%)
18 19 20 21 22 23 24 25 26 27 28 29 30
A
A-B
A&B
B-A
B
Probability
1.0
0.5
0.0
1 2 3 4 5
-5 -4 -3 -2 -1
Nucleotide position (5′ ↔ 3′)
C
Genomic origin (%)
Repeat (+)
Repeat (-)
Intergenic
Exon (+)
Exon (-)
Intron (+)
Intron (-)
tRNA
rRNA
Others
Proportion of each length class (%)
18 19 20 21 22 23 24 25 26 27 28 29 30
A
A-B
A&B
B-A
B
Probability
Nucleotide position (5′ ↔ 3′)

Supplementary Figure 12. Venn diagram of Dicer-independent reads and MoERI-1-dependent reads using distinct and total sRNAs. (A) Venn diagram of the proportions of distinct and total reads in the WT using Dicer-independent and MoERI-1-dependent reads. Genomic origin, length distribution, and nucleotide composition were investigated using the distinct **(B)** and total **(C)** reads**.**
